# *In vitro* activity profiles and temporal trends in reduced susceptibility to azoles in *Coccidioides* in the United States

**DOI:** 10.1128/aac.00744-26

**Published:** 2026-09-03

**Authors:** Nathan P. Wiederhold, Charlotte Bisson, David A. Stevens, Chiung-Yu Hung, Jose Lopez-Ribot, Thomas F. Patterson, George R. Thompson III

**Affiliations:** 1 Fungus Testing Laboratory, Department of Pathology and Laboratory Medicine, University of Texas Health Science Center at San Antonio 14742, San Antonio, Texas, USA; 2 University of California 1438, Berkeley, California, USA; 3 Division of Infectious Diseases and Geographic Medicine, Department of Medicine, Stanford University School of Medicine 10624, Stanford, California, USA; 4 Department of Molecular Microbiology and Immunology, South Texas Center for Emerging Infectious Diseases, University of Texas at San Antonio 843937, San Antonio, Texas, USA; 5 Department of Medicine, Division of Infectious Diseases, University of Texas Health Science Center at San Antonio 171806, San Antonio, Texas, USA; 6 Department of Internal Medicine, Division of Infectious Diseases and the Department of Medical Microbiology and Immunology, University of California-Davis Medical Center 21772, Sacramento, California, USA; 7 University of California Davis Center for Valley Fever, Sacramento, California, USA; University Children's Hospital Münster, Münster, Germany

**Keywords:** *Coccidioides*, coccidioidomycosis, fluconazole, itraconazole, isavuconazole, posaconazole, voriconazole, antifungal susceptibility testing, endemic mycoses

## Abstract

Treatment of symptomatic patients with coccidioidomycosis often involves the use of triazole antifungals. There is concern for reduced susceptibility of *Coccidioides* to fluconazole, as a high percentage of isolates were previously reported to have elevated fluconazole MICs. We reviewed our recent azole MICs against *Coccidioides* and examined temporal trends over a 20-year period. Our clinical laboratory database was queried for data against *Coccidioides* between 2006 and 2025, and trends in GM MICs and the percentage of isolates with elevated MICs to the azoles were assessed. All testing had been performed by broth dilution according to Clinical and Laboratory Standards Institute (CLSI) M38 methods. The results over the 10-year period from 2016 to 2025 were compared to those previously reported by our group from 2001 to 2015. Overall, the results between the two periods showed similar MIC parameters and distributions for fluconazole, itraconazole, posaconazole, and voriconazole, with posaconazole demonstrating the most potent activity, while fluconazole demonstrated reduced susceptibility. Isavuconazole MIC parameters were available during the second period and similar to those of voriconazole. Weak correlations were observed between fluconazole MICs and those of the other azoles. Fluctuations in GM MICs and the percentage of isolates with elevated MICs were observed with fluconazole, itraconazole, and posaconazole, with elevated values observed between 2011 and 2019. Continued surveillance is needed, and outcome data are required to determine if the consistently reduced susceptibility observed with fluconazole or in those infected with strains that have higher MICs results in poorer outcomes in patients with coccidioidomycosis.

## INTRODUCTION

Coccidioidomycosis is an invasive fungal infection caused by *Coccidioides immitis* and *C. posadasii*. These dimorphic fungi exist as mycelia within the soil. During periods of low precipitation, arthroconidia (e.g., spores) develop and, following aerosolization, may be inhaled with resultant infection (1, 2). Treatment of symptomatic patients with coccidioidomycosis consists of triazoles (fluconazole, itraconazole, voriconazole, posaconazole, isavuconazole) and/or an amphotericin B formulation (3).

Antifungal therapy has significantly and favorably impacted patient outcomes, particularly among those with severe forms of coccidioidomycosis. Despite the availability of these agents, some patients develop progressive disease refractory to currently available treatment options. Treatment failure may be secondary to an insufficient host immune response, suboptimal pharmacokinetic/pharmacodynamic parameters (4), or elevated mean inhibitory concentrations (MICs), reducing drug susceptibility. Species-specific clinical breakpoints and/or epidemiologic cutoff values (ECVs) have not been established for *Coccidioides*, thereby complicating the interpretation of MIC values. Previous work from our group reported elevated MICs for fluconazole against *Coccidioides* species (>16 µg/mL in 37% of isolates) (5). As these previous results were published nearly 10 years ago (data from the period of 2001 to 2015), we sought to evaluate more recent *in vitro* susceptibility data for the triazoles against *Coccidioides* species. We also assessed temporal trends in MIC patterns over a 20-year period.

## MATERIALS AND METHODS

### Isolates

The clinical antifungal susceptibility database in the Fungus Testing Laboratory at the University of Texas Health Science Center at San Antonio was reviewed for records of *Coccidioides* spp. received for testing between January 2001 and December 2025, which were included. For trends in geometric mean (GM) MIC values, data from 2001 to 2005 were excluded due to the small number of isolates tested for azoles other than fluconazole. All isolates were subcultured onto potato flake agar (PFA) on the day of receipt, prior to testing. Species identifications had been made by the submitting clinical microbiology laboratories or by the fungus testing laboratory by phenotypic characteristics, a previously available chemiluminescent DNA probe (AccuProbe, Gen-Probe, Hologic), or DNA sequence analysis of the ITS and D1/D2 regions (6). Data on the states from which isolates were received were also collected. The number of coccidioidomycosis cases reported to the Centers for Disease Control and Prevention National Notifiable Diseases Surveillance System (CDC NNDSS) each year from 1998 to 2023 were also reviewed, as were those available from the California Department of Public Health and Maricopa County, Arizona, for the years 2024 and 2025.

### Antifungal susceptibility testing

Antifungal susceptibility testing was performed by broth macrodilution testing according to the methods described in the Clinical and Laboratory Standards Institute (CLSI) M38 standard as part of clinical diagnostic testing (7). Stock solutions of fluconazole, itraconazole, posaconazole, voriconazole, and isavuconazole (Sigma-Aldrich, St. Louis, MO) were prepared at 100× in dimethyl sulfoxide (DMSO) with further dilutions prepared in RPMI-1640 (0.165M MOPS, pH 7.0, 0.2% glucose, without bicarbonate), such that the final DMSO concentration within the *in vitro* assay was 1% v/v. Minimum inhibitory concentrations (MIC) of posaconazole, voriconazole, itraconazole, and isavuconazole were read at 100% inhibition of growth compared to drug-free control after 48 h of incubation at 35°C, and fluconazole MICs were measured at 50% growth inhibition.

### Data analysis

All data were assessed by descriptive statistics, including MIC ranges, MIC values at which 50, 90, 97.5, and 99% of the isolates were inhibited (MIC_50_, MIC_90_, MIC_97.5_, and MIC_99_, respectively), geometric mean (GM) MIC, and modal MIC values. Following log_2_ transformation of individual MIC values, differences in GM MICs were assessed for significance by analysis of variance (ANOVA) with Dunnett’s post-test comparing each group to control (fluconazole). MIC values greater than the highest concentration tested were assigned a value one dilution higher for statistical purposes, while those at the lowest concentration range were left unchanged. A *P*-value of <0.05 was considered statistically significant. The correlations between different azole MICs were assessed by Pearson correlation using log_2_-transformed MIC values.

## RESULTS

### *In vitro* susceptibility profile of azoles: 2016 to 2025 vs. 2001–2015

MIC parameters for the azoles over the last 10 years are shown in Table 1, along with those we have previously published with data obtained between 2001 and 2015 (5). Overall, the MIC ranges, MIC_50_, and MIC_90_ values were similar between the two periods. Although the GM MIC values for fluconazole, itraconazole, and voriconazole were statistically higher during the last 10 years (*P* ≤ 0.05 for each comparison), the values were within a 1.14- to 3.27-fold range of the previous period. In contrast, the GM MIC for posaconazole between 2016 and 2025 was just under half of that observed for the previous period. Posaconazole also demonstrated the most potent *in vitro* activity over the last 10 years, as evident by the lowest GM MIC value. In contrast, voriconazole had the most potent activity during the period of 2001 to 2015, but this GM MIC increased by over threefold during the past 10 years. We also report the MIC parameters for isavuconazole over the last 10-year period. The isavuconazole GM MIC value was similar to that observed with voriconazole but higher than that of posaconazole. However, no comparison between isavuconazole MIC parameters or those of the other azoles can be made with the previous period, as this azole was not used clinically or tested for activity against *Coccidioides* during this time.

**TABLE 1:** Azole minimum inhibitory concentration (MIC) profiles against clinical isolates of *Coccidioides* species cultured from patients in U.S. institutions between 2001 to 2015 and 2016 to 2025^*a*^

MIC parameters	Fluconazole	Itraconazole	Voriconazole	Posaconazole	Isavuconazole
2001–2015 (previously published)					
Number	581	486	499	377	---
Range	01.25–>64	≤0.03–>16	≤0.03–8	≤0.03–>16	---
MIC_50_	8	0.25	0.125	0.125	---
MIC_90_	16	0.5	0.25	0.25	---
MIC_97.5_	64	1	1	0.5	---
MIC_99_	64	4	2	1	---
GM MIC	7.77	0.245	0.107	0.141	---
Mode	8	0.5	0.125	0.25	---
2016-2025					
Number	1643	1256	1468	1247	782
Range	0.25 –>64	≤0.03–>16	≤0.03–4	≤0.03–1	≤0.03–8
MIC_50_	8	0.125	0.125	0.06	0.25
MIC_90_	16	0.5	0.25	0.25	1
MIC_97.5_	64	1	0.5	0.5	2
MIC_99_	>64	1	1	0.5	2
GM MIC	8.85	0.427	0.350	0.076	0.332
Mode	8	0.125	0.125	0.06	0.25

^*a*^ MIC_50_, MIC_90_, MIC_97.5_, and MIC_99_, MIC values at which 50, 90, 97.5, and 99% of the isolates were inhibited, respectively; GM MIC, geometric mean MIC; ---, not available for Isavuconazole for 2001-2015.

**Fig 1:**
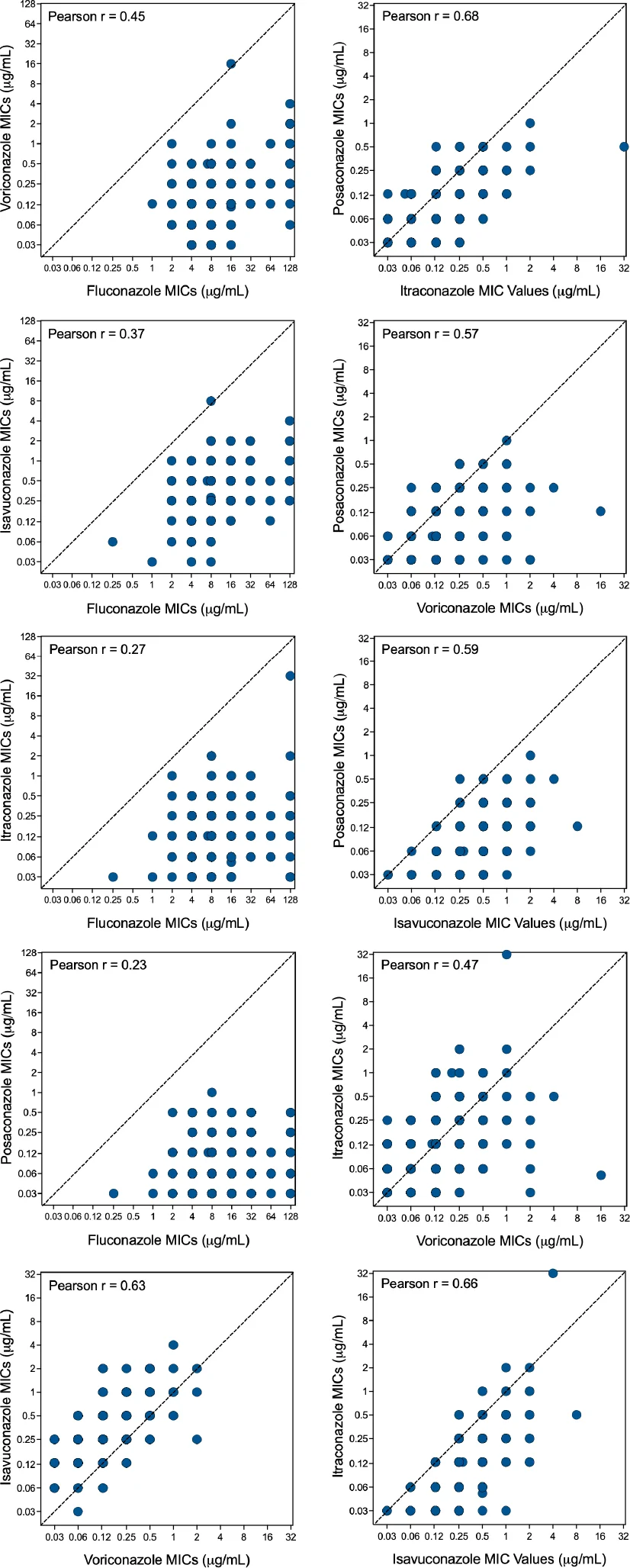
MIC comparisons between the different azoles against *Coccidioides* spp. clinical isolates. MICs were measured after 48 h of incubation at 35°C as the lowest concentration that resulted in 100% inhibition of growth for isavuconazole, itraconazole, posaconazole, and voriconazole, and 50% inhibition of growth compared to the drug-free growth control for fluconazole. The diagonal lines split each of the graphs between the two antifungals that are shown, with points to the left of the line representing higher MIC values for the antifungal on the *y*-axis and those to the right of the line representing higher MIC values for those on the *x*-axis.

Pairwise comparisons were also made between triazoles for which MIC values were available for each of the two agents being compared (Fig. 1). Good correlations (Pearson correlation coefficients *r* > 0.6) were observed for the pairs of voriconazole/isavuconazole, isavuconazole/posaconazole, isavuconazole/itraconazole, and itraconazole/posaconazole. In contrast, correlations between fluconazole and all other triazoles were weak (*r* = 0.23–0.45).

### Upper limit of normal MIC values

As no breakpoints are available for any antifungal against *Coccidioides* species, and calculation of epidemiologic cut-off values (ECV) was not possible since all data came from a single laboratory, we determined the upper limit of normal (ULN) for each azole by calculating the MIC values that inhibited 97.5 and 99% of all isolates (MIC_97.5_ and MIC_99_, respectively) (Table 1). For each of the azoles, the MIC_97.5_ was the same for fluconazole (≥64 µg/mL), itraconazole (1 μg/mL), and posaconazole (0.5 μg/mL) between the two time periods. For voriconazole, the MIC_97.5_ was one dilution lower during the last 10 years (0.5 μg/mL for 2016 to 2025) compared to our previously published results (1 μg/mL). MIC_99_ values for the period of 2016 to 2025 were one dilution lower for itraconazole, voriconazole, and posaconazole compared to the previous period, but were at least one dilution higher for fluconazole (>64 µg/mL vs. 64 μg/mL, respectively).

### Trends in geometric mean MICs and percentage of isolates with elevated azole MICs over time

**Fig 2:**
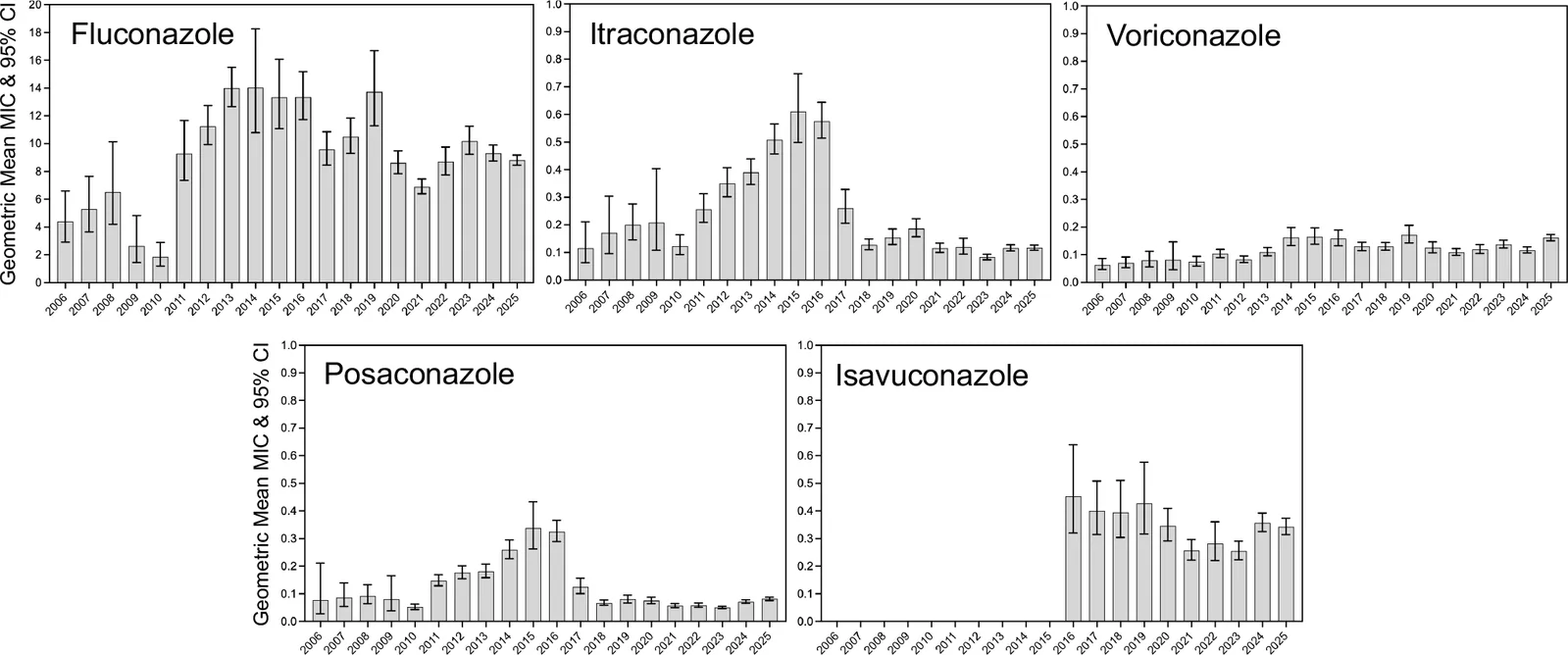
Changes in geometric mea (GM) MICs for the azoles over time (2006 to 2025). GM MICs and 95% confidence intervals (CI) are presented for each year for which there were at least 10 MIC values for each antifungal.

**Fig 3:**
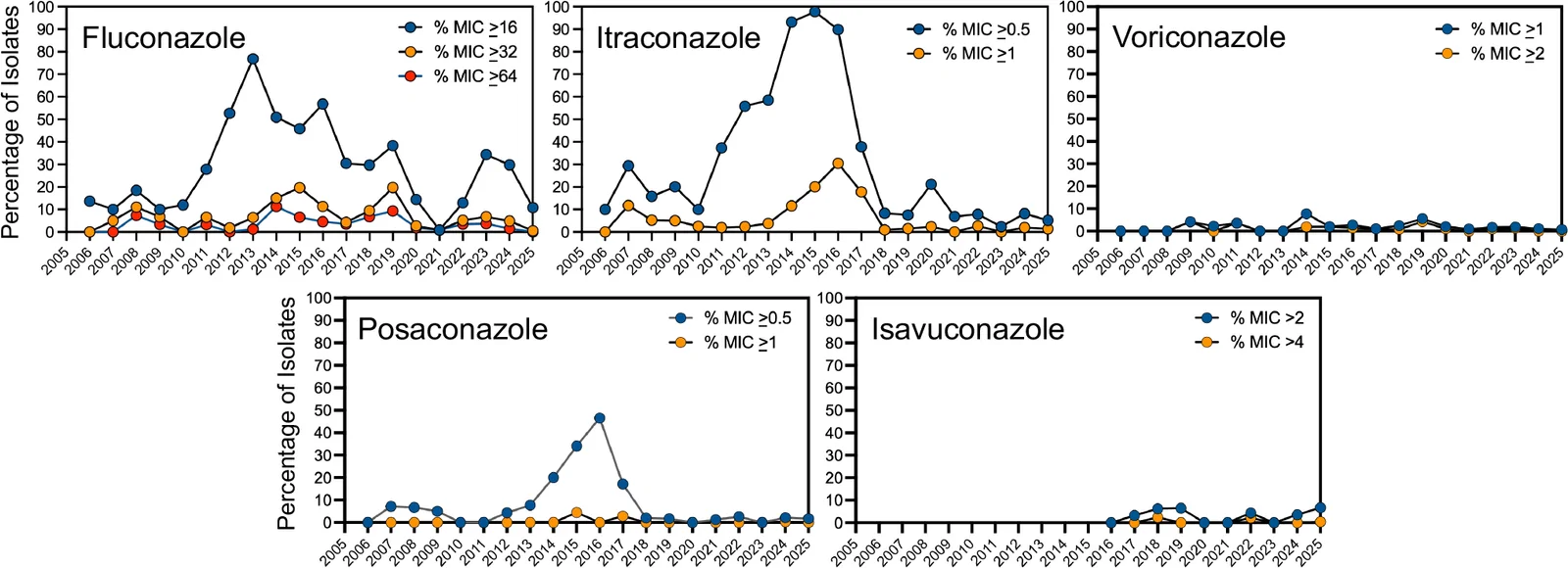
Percentage of isolates demonstrating elevated MICs to the azoles between 2006 and 2025. For fluconazole, the percentages of isolates with MICs at ≥16, ≥32, and ≥64 µg/mL are shown. For itraconazole and posaconazole, the percentages of isolates with MICs at ≥0.5 and ≥1 µg/mL are shown. For voriconazole, the percentages of isolates with MICs at ≥1 and ≥2 µg/mL are shown. For isavuconazole, the percentages of isolates with MICs at ≥2 and ≥4 µg/mL are shown.

We also assessed temporal trends in the GM MIC values and percentage of isolates with elevated MICs to the different azoles over time (Fig. 2 and 3). For fluconazole, between 2011 and 2019, GM MICs were elevated, and the percentage of isolates with elevated MICs (i.e., ≥16 µg/mL) was 30% or greater (range 30 to 77%) and sharply increased between 2011 and 2013, reaching 77%, before beginning to decline in 2021 and later again increasing to 34% in 2023. The percentage of isolates with fluconazole MICs ≥ 64 µg/mL, the highest concentration tested, ranged from 0% to as high as 11%, with greater than 5% having MIC values at or above this value from 2014 to 2015 and again between 2018 and2019.

Interestingly, we also observed increases in the GM MICs and the percentage of isolates with elevated itraconazole and posaconazole MICs (i.e., ≥0.5 µg/mL for both azoles) between 2011 and 2017 and 2014 to 2016, respectively. For itraconazole, this was >30% of isolates during this period, reaching as high as 98% in 2015. Similarly, the number of isolates with itraconazole MICs ≥ 1 µg/mL, corresponding to the MIC_97.5_ for both study periods, was ~30% in 2016. For posaconazole, the number of isolates with MIC values ≥ 0.5 µg/mL ranged from 12 to 30% of isolates during this period. Interestingly, a similar trend was not observed for voriconazole. Of the isolates with elevated fluconazole MICs ≥ 16 µg/mL, 41.3% were received from institutions in California and 14.6% from institutions in Arizona. Of those from California, most were from institutions located in the San Francisco Bay area and Central Valley. Similar results were observed for isolates with fluconazole MICs ≥ 32 µg/mL (38.3 and 13.6% from California and Arizona, respectively). However, the true geographic distribution of isolates with elevated triazole MICs is unknown, as ~25% was received from national commercial laboratories, and the location from which these strains originated is not available.

**Fig 4:**
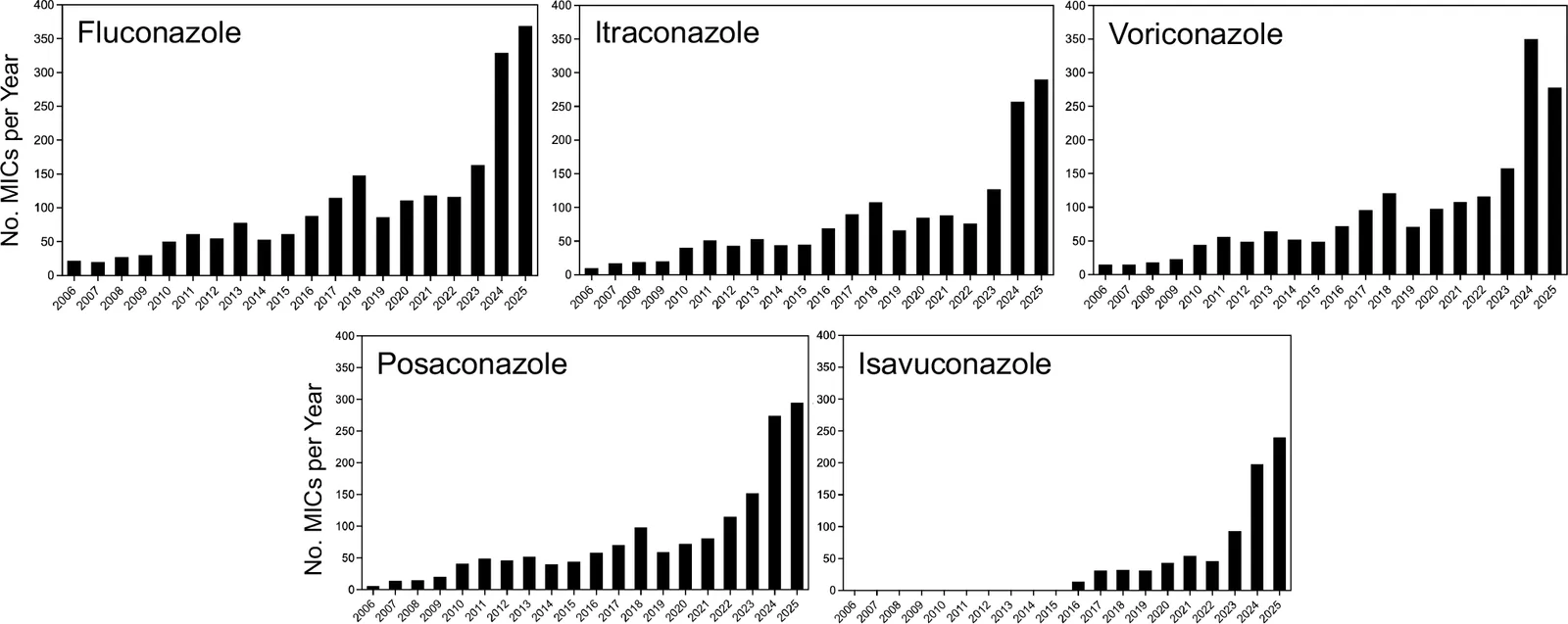
Number of MIC tests performed for each azole each year from 2006 to 2025.

**Fig 5:**
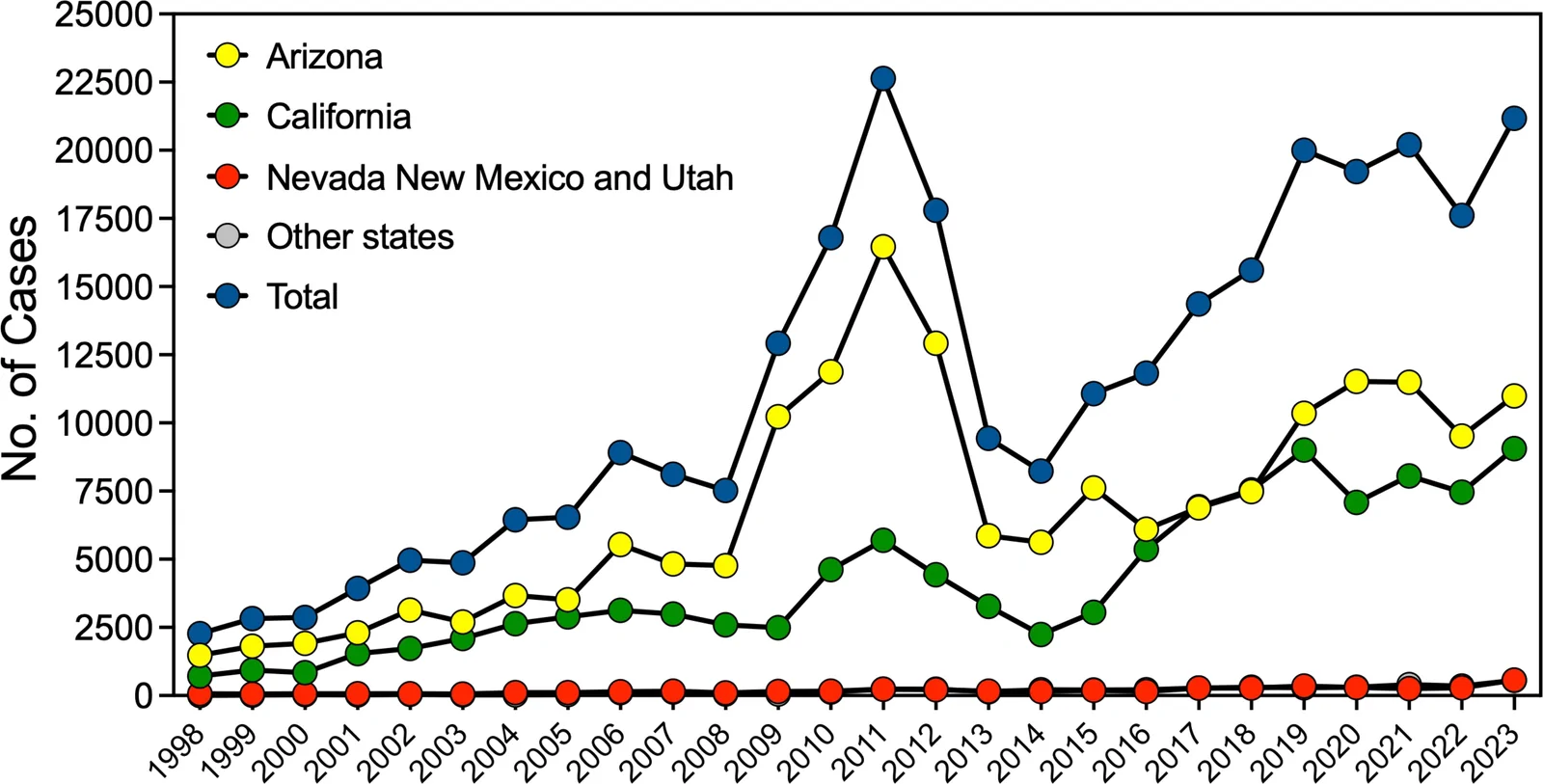
Number of reported cases of coccidioidomycosis, overall and per state, from 1998 to 2023. Data were obtained from the Centers for Disease Control and Prevention statistics on coccidioidomycosis.

The number of MIC tests performed for each azole per year is shown in Fig. 4. Overall, the number of susceptibility tests performed for each drug slowly trended upwards until recently when we observed sharp increases for testing each antifungal agent in 2024 and 2025. Of note, the number of cases of coccidioidomycosis reported to the CDC NNDSS began to increase a few years before the increase in the percentage of isolates with elevated azole MICs was observed (8). As shown in Fig. 5, there was a marked increase in the number of cases beginning in from 2008 to 2011 (~3 fold), primarily observed in Arizona, before a similar sharp decrease in cases was observed from 2012 to 2014. Since 2014, the number of cases of coccidioidomycosis has steadily increased. Although final data for 2024 and 2025 are not yet available from the CDC NNDSS, provisional data from the California Department of Public Health and Maricopa County, Arizona, for these 2 years suggest that the number of cases remains elevated (9, 10).

## DISCUSSION

The incidence of coccidioidomycosis continues to increase, with the majority of cases within the endemic regions of CA and AZ, although up to 25% of cases may be diagnosed outside the traditionally endemic regions. Outbreaks are known to occur after dust storms, earthquakes, and wildfires (11–14). Coincident with the increase in total cases, there is also concern that the endemic area within the U.S. is expanding and may be related to ongoing climate change (15, 16). Patients who are infected may be asymptomatic or develop a mild, self-resolving respiratory illness, and the majority of those with acute respiratory disease resolve with or without antifungal treatment (3). However, a small percentage will develop more severe forms of the disease and require antifungal therapy, as do immunosuppressed patients and those with cardiopulmonary comorbidities. Many of these individuals are treated with a triazole antifungal that can be administered orally. However, there is concern about reduced susceptibility of *Coccidioides* clinical isolates to fluconazole (5). During the period of 2001 to 2015, we observed that elevated fluconazole MICs (≥16 µg/mL) were observed in over a third of isolates (37.2%), and 3.8% of isolates had extremely high fluconazole MICs (≥64 µg/mL) (5).

In the current study, we expand upon the *in vitro* susceptibility profiles of fluconazole and the mold-active azoles itraconazole, voriconazole, and posaconazole against *Coccidioides* isolates by showing results for the last 10 years (2016 to 2025). In addition, we also now provide isavuconazole MIC results, which were not available for the previous period. Posaconazole had the most potent *in vitro* activity, followed by voriconazole, isavuconazole, and itraconazole, and, lastly, fluconazole, as demonstrated by GM MIC values. Overall, the MIC parameters for posaconazole, voriconazole, itraconazole, and fluconazole did not change markedly between the two periods (2001–2015 and 2016–2025). However, MIC values for fluconazole did not correlate strongly with those of the other azoles, while moderate correlations were observed between the other agents.

We have observed marked fluctuations in GM MICs and the percentage of isolates with elevated MICs to fluconazole, itraconazole, and posaconazole, with elevated rates of reduced susceptibility for each agent occurring within a similar time period (2011 to 2019). The reasons for these fluctuations are unclear. The number of isolates tested each year during this period was relatively consistent with a slight upward trend. Laboratory variability is unlikely, as the methodology used for *in vitro* susceptibility testing, the sources from which materials were acquired, and the personnel performing the assay remained consistent throughout this study. Interestingly, the shift to higher GM MIC values and percentage of isolates with elevated MICs appeared to occur a year or two after an uptick in national cases, as reported by the CDC NNDSS. It is unknown if the changes in the number of cases and changes in MICs for these three azoles are related. As the CDC NNDSS and more recent data from California and Maricopa County, Arizona, demonstrate an increase in the number of coccidioidomycosis cases over the last few years, it will be interesting to see if we observe another upswing in GM MIC values and the number of isolates with higher MICs over the next few years. Unsurprisingly, most isolates with elevated azole MICs were cultured from patients in California and Arizona since the majority of isolates were received from these two states.

With the exception of 2021, fluconazole GM MICs have been above 8 μg/mL since 2010 and MIC_90_ values at or above 16 μg/mL. The fluconazole ULN MIC values (i.e., MIC_97.5_ and MIC_99_) were at or above the highest concentration tested (64 μg/mL). This activity of fluconazole against *Coccidioides* is somewhat analogous to what is observed with this azole against *Candida glabrata*, for which CLSI has classified all strains with MIC values of ≤32 µg/mL as susceptible-dose-dependent. While fluconazole can be used to treat certain patients with *C. glabrata* infections, higher doses are often used. Higher doses of fluconazole are also, at times, used to treat patients with coccidioidomycosis, including those with CNS disease (3). However, breakpoints are not currently available for fluconazole or any other antifungal against *Coccidioides* spp. Neither clinical data correlating outcomes with MIC results nor pharmacokinetic/pharmacodynamic data from experimental models are available. In addition, ECVs could not be calculated since these data were from a single laboratory, and results from at least three independent laboratories are required, and more than 50% of the data cannot come from one laboratory (17, 18). Very few clinical microbiology laboratories offer *in vitro* susceptibility testing against *Coccidioides* spp.

Overall, these results demonstrate that there have not been major changes in *in vitro* susceptibility patterns for the azoles during the last 10 years. Fluctuations in azole GM MICs and the percentage of isolates with reduced susceptibility occur over time, especially for fluconazole. However, as this was not a systematic population surveillance study, but rather a large data set from our clinical reference laboratory, the true prevalence of *Coccidioides* isolates with elevated MICs to fluconazole and the other azoles is unknown. In addition, we did not exclude results from subsequent isolates collected from the same patients. As noted previously, breakpoints are not available for any antifungal against *Coccidioides* species, and outcome data are needed to determine if the consistently reduced susceptibility observed with fluconazole or in those infected with strains that have higher MICs with other azoles results in poorer outcomes in patients with coccidioidomycosis. Genus-level identification was only available for the majority of isolates. Thus, we were not able to determine if differences in azole MIC patterns exist between *C. immitis* and *C. posadasii*.
